# CSF/serum quotient graphs for the evaluation of intrathecal C_4 _synthesis

**DOI:** 10.1186/1743-8454-6-8

**Published:** 2009-07-02

**Authors:** Barbara Padilla-Docal, Alberto J Dorta-Contreras, Raisa Bu-Coifiu-Fanego, Alexis Rodriguez Rey

**Affiliations:** 1 Central Laboratory of Cerebrospinal Fluid (LABCEL) Faculty of Medical Sciences "Dr Miguel Enriquez" Superior Institute of Medical Sciences of Havana AP 10049, 11000 CP Havana City, Cuba

## Abstract

**Background:**

Cerebrospinal fluid (CSF)/serum quotient graphs have been used previously to determine local synthesis in brain of immunoglobulins and C_3 _complement component. The aim of this study was to use the same technique to construct quotient graphs, or Reibergrams, for the beta globulin C_4 _and to evaluate the method for assessing intrathecal synthesis in neurological disease.

**Methods:**

The constants in the previously-defined Reibergram for immunoglobulin IgA were used to calculate the CSF/serum quotient for C_4_. CSF and serum were analyzed for C_4_, IgA and albumin from a total of 12 patients with meningoencephalitis caused by encapsulated microorganisms and 10 subjects without infections or inflammatory neurological disease, some of which had dysfunction of the blood-CSF barrier,

**Results:**

The formula and C_4 _Reibergram with the constants previously found for IgA, determined the intrathecal C_4 _synthesis in CSF. The intrathecal C_4 _fraction in CSF (C_4 _loc in mg/l) was compared to the C_4_-Index (fraction of CSF: serum for C _4_/fraction of CSF: serum for albumin). There was a significant correlation between the two formulae. The CSF/Serum quotient graph was superior for detecting intrathecal synthesis of C_4 _under variable conditions of blood-CSF barrier permeability.

**Conclusion:**

The C_4 _Reibergram can be used to quantify the intrathecal synthesis of this component of the complement system in different infectious diseases of the central nervous system and is especially useful for patients with blood-brain barrier dysfunction.

## Background

Cerebrospinal fluid (CSF) analysis has great potential for the diagnosis of neurological diseases. Reibergrams or CSF/serum quotient graphs [1,2] are diagrams that analyze in an integrated way both the function of the blood-CSF barrier and intrathecal protein synthesis, to aid in the diagnosis of central nervous system (CNS) diseases associated with specific patterns of immunoglobulin response. This diagram was first defined for the major classes of immunoglobulins [2,3] empirically based on the results of thousands of profiles and was subsequently confirmed by application of Fick's law of diffusion in the theory of molecular diffusion/flow rate [4]. Fick's law states that the diffusion of a protein through a barrier depends on the diffusion coefficient of the molecule and on the concentration gradient between the compartments on either side of the barrier. The diffusion coefficient depends on the molecular size of the protein. Fick's second law shows how the local concentration gradient is nonlinearly modified by variations in the CSF flow rate. The theory of molecular diffusion/flow rate of CSF explains that the diffusion of proteins through the blood/CSF barrier has a hyperbolic distribution and is able to explain the physiological and pathophysiological dynamics of proteins in the CNS. This has permitted its use for other immunoglobulins such as IgG_3 _[5], IgE [6] and more recently C_3c _[7].

The complement system is one of the humoral mediators of immunity and inflammation; it is formed by more than thirty plasma and membrane proteins. Raised levels of C_4_, one of the components, may be found in the CSF in lupus meningoencephalitis [8,9], in progressive supranuclear palsy [10] and other neurological diseases [11] such as autism [12], schizophrenia [13], cerebral parenchymal cysticercosis [14], meningoencephalitis [15] and brain tumors [16]. Cytokines associated with amyloid plaques in Alzheimer's disease stimulate human glial and neuronal cell cultures to secrete early complement proteins [17,18]. Some of these proteins are bound to immunoglobulins or to components of the cellular membrane [19], although they normally circulate in the form of pro-enzymes with a latent pro-enzyme activity.

C_4 _protein is a beta globulin with a sedimentation coefficient of 18.7, molecular weight 200 kDa and a serum concentration of 430 μg/mL. It is activated by the complement system and is a C_2 _receptor. C4a acts as anaphylatoxin similar to C_3a_but is weaker, while C_4b _acts as an opsonin promoting phagocytosis by binding to complement receptors. It is important to assess C_4 _intrathecal synthesis if the classical complement pathway has been activated in infectious and autoimmune neurological diseases. Combined with analysis of C_3c _intrathecal synthesis [20,21], this knowledge is needed to understand the pathophysiology of these disorders.

The aim of this paper was to construct quotient graphs (Reibergrams) for the beta globulin C_4 _and to determine the intrathecal synthesis of C_4 _in patients with bacterial meningoencephalitis. To do this we used IgA which has molecular characteristic similar to the C_4 _molecule and its previously-defined CSF/serum quotient graph, to create the formula and Reibergram for C_4_.

## Methods

### Patients

Twelve patients with meningoencephalitis caused by encapsulated microorganisms were studied: nine patients suffering from *Streptococcus pneumoniae *and three with *Haemophilus influenzae *(mean age of 32.5 years, range 3 to 46 years). A control group consisted of ten subjects without infections or inflammatory neurological disease and that did or did not have dysfunction of the blood-CSF barrier (mean age 17 years, range 9 months to 48 years), The research was approved by the Committee on Bioethics Research of the Faculty of Medical Sciences, Dr. Miguel Enriquez Superior Institute of Medical Sciences of Havana, Havana City, Cuba. All patients and persons whose samples were used as controls, and parents or tutors of the children gave their informed consent to carry out a diagnostic lumbar puncture.

### CSF and serum samples

CSF samples were obtained by lumbar puncture and simultaneous blood samples were taken to obtain serum. Cells were removed from CSF by centrifugation and samples with blood contamination were eliminated. Control samples were from CSF and serum collections held in our laboratory from patients with suspicion of meningoencephalitis but were subsequently found to have febrile convulsions without biological agents or verified autoimmune causes. Aliquots of both patient and control samples were kept at -70°C for up to 30 days after the puncture date before analysis.

### Analysis of CSF and serum

C_4_, IgA and albumin were quantified by radial immunodiffusion with NOR PARTIGEN^© ^and LC PARTIGEN^© ^plates (Dade Behring, Marburg, Germany) for serum and CSF, respectively. Radial immunodiffusion detects the reaction of antigen and antibody by a precipitation reaction based on the principle that a quantitative relationship exists between the amount of antigen placed in a well cut in the agar-antibody plate and the resulting ring of precipitation. This end point method requires that the precipitation rings reach the maximal possible size, which often requires 48–72 h of diffusion. A standard curve was experimentally determined with known standards and used for the determination of protein concentration corresponding to any diameter size.

### C_4 _Reibergram and index

The CSF/Serum quotient graph or Reibergram for C_4 _was created taking into consideration the molecular characteristics of this protein. In addition, the C_4_-Index was calculated using the following formula [22]:



As a cut off for discrimination between a normal and pathological C_4_-Index, we used C_4_-Index = 0.46

Among proteins found in CSF and serum, C_4 _is most similar to IgA in terms of molecular characteristics (Table 1). The constants used for the creation of the formula for intrathecal C_4 _detection were those for IgA [3] which are likely to be similar to C_4 _according to Fick's law of diffusion. Hence the Reiber's quotient for C_4 _was calculated using the following formula:

**Table 1:** Molecular characteristics of immunoglobulins and complement proteins

	IgG_1_	IgA	IgM	C_3c_	C_4_
Molecular Mass (kDa)	150	160	900	145	200

Hydrodynamic radius (nm)	52	58	270	54	65



Where Q C_4 _(lim) is the upper discrimination line of the reference range, Q C_4 _= C_4 _(CSF)/C_4 _(Serum), a = intercept hyperbole value on the y axis, b = x value to asymptotic hyperbole line, c = constant and a/b = hyperbole slope. The following values previously obtained for IgA were used: a/b = 0.77, b^2 ^= 23 × 10^-6^, c = 3.1 × 10^-3^.

Thus the formula for the amount of locally-synthesized C_4 _in the CSF or C_4 _(loc) is:



The program MEDCALC version 6.0 was used to perform the statistical comparison.

## Results

**Figure 1:**
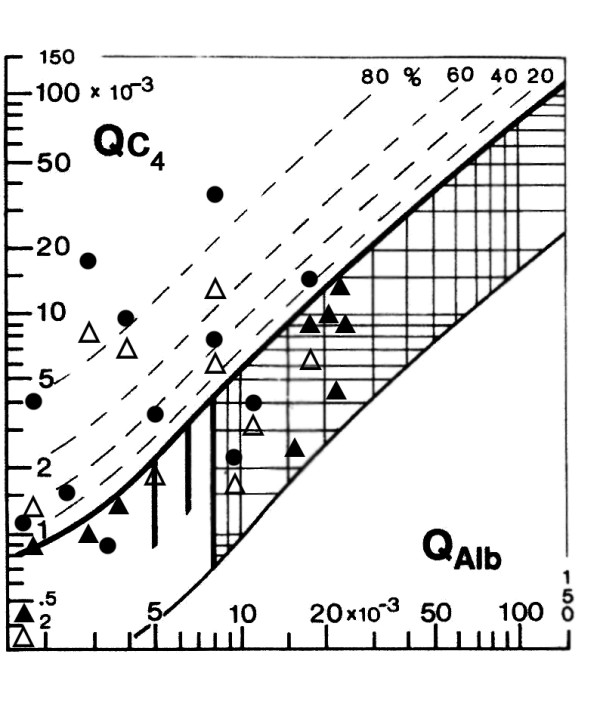
**CSF/serum quotient diagram for C_4 _(Reibergram)**. The upper hyperbolic curve (thick line) represents the discrimination line between brain-derived and blood-derived protein. Values above this upper line represent intrathecal C_4 _synthesis. The dashed hyperbolic lines indicate the extent of intrathecal synthesis as intrathecal fractions with 20, 40, 60, and 80% of the measured total immunoglobulin concentration in CSF, with reference to the discrimination line as 0% intrathecal synthesis. The limit of the reference range for QAlbumin between normal and increased CSF protein concentrations due to blood-CSF barrier dysfunction is indicated by the age-dependent vertical lines at Q Albumin 5.5 × 10-3 (up to 15 years), at QAlbumin 6.5 × 10-3 (up to 40 years), and at Q Albumin 8 × 10-3 (up to 60 years). Nine of the patients with neurological infectious meningoencephalitis (Black circle) had values above the upper solid line which indicate intrathecal synthesis of C_4_. Ten controls (Black triangle) without infectious neurological disorders had values in the normal range, indicating no intrathecal C_4 _synthesis. Also plotted are QIgA (White triangle) from 10 patients suffering from meningoencephalitis and five of these had IgA intrathecal synthesis. Two patients had no detectable IgA in CSF.

The Q C_4 _quotient graph for the patients and controls under study with a range of values of Q albumin (QAlb) is shown in figure 1. The Q C_4 _values for all twelve patients with meningoencephalitis are above the lower thin hyperbolic line. For nine of these twelve patients Q C_4 _was located above the upper solid line showing that these patients synthesized C_4 _intrathecally. The Q C_4 _for all control patients is in the area between the thick hyperbolic line and the lower thin line, indicating that there is no CNS synthesis of this component. Also included is the QIgA of the patients with inflammatory meningoencephalitis and only five of these are in the region that indicates intrathecal synthesis. Another two patients had no detectable IgA in CSF. Hence the pattern of intrathecal synthesis of IgA is not the same as for C_4_, suggesting that IgA has a different role in the immune response, despite the similar molecular flux from blood to CSF according to the molecular size. Mean values, range and coefficient of variation for C_4 _and IgA in serum and CSF are shown in Table 2.

**Table 2:** Mean value, range and coefficient of variation (CV) in CSF and serum for C_4_and IgA

		Meningoencephalitis	Controls
Size (n)		12	10

CSF C_4 _(mg/L)	Mean	12.07	2.43
	
	Range	0.17–114	0.17–12
	
	CV	2.67	1.60

Serum C_4 _(g/L)	Mean	1.12	1.08
	
	Range	0.19–5.6	0.37–5.6
	
	CV	0.71	1.56

Q C_4 _× 10^3^	Mean	7.35	1.49
	
	Range	1.1–39.4	0.38–10.8
	
	CV	1.45	1.79

Q Alb × 10^3^	Mean	5.91	5.36
	
	Range	0.3–17.1	0.3–27.41
	
	CV	0.79	0.99

C_4_-Index	Mean	1.91	0.74
	
	Range	0.3–5.05	0.02–3.66
	
	CV	0.96	1.49

CSF IgA (mg/L)	Mean	1.09	1.28
	
	Range	0.17–4.62	0.32–5.6
	
	CV	1.17	1.37

Serum IgA (g/L)	Mean	1.12	0.67
	
	Range	0.32–5.6	0.17–1.7
	
	CV	1.38	0.90

QIgA × 10^3^	Mean	1.76	0.92
	
	Range	0.16–10.62	0.16–3.5
	
	CV	1.05	1.34

Notes:

Q = quotient (CSF/Serum)

Alb = Albumin

C_4_-Index = (C_4 _CSF/C_4 _serum)/(Albumin CSF/Albumin serum)

Hitherto, the formula used to quantify the intrathecal synthesis of C_4 _complement has been the C_4_-Index [22,23]. In this study, the results obtained from the CSF/Serum quotient formula were compared to C_4_-Index values. Using C_4_-Index, among the non-inflammatory controls, there were 2 false positives, although intrathecal complement synthesis should not occur in such cases. With C_4_-Index > 0.46 we find in 10/12 case of meningoencephalitis a pathological increase, i.e. an intrathecal synthesis (Table 3).

**Table 3:** Comparison of the results for intrathecal fraction of C_4 _in CSF calculated as C_4 _loc (mg/l) and as the C_4_-Index in patients with inflammatory disease and non-inflammatory controls.

	Intrathecal C_4 _fraction		C_4_-Index	
	C_4 _loc > 0	C_4 _loc < 0	> 0.46	< 0.46

Meningoencephalitis	9/12	3/12	10/12	2/12

Non inflammatory controls	0/10	10/10	2/10*	8/10

The discrimination cut off for the C_4_-Index = 0.46.

* Two false positives

There was a positive correlation between the C_4_-Index and the CSF/Serum quotient graph for C_4_, r = 0.7287 and range 0.4432 – 0.8800, for a confidence interval of 95%, *p *= 0.0001. The general sensitivity of C_4_-Index, i.e. the probability that this result will be positive when the inflammatory process is present, was only 66% of true positive rate. The C_4_-Index general specificity was 85%, meaning that the probability that the C_4_-Index will be negative when the inflammatory process is not present was 85% of true negative. The positive predictive value or the probability that the inflammatory process is present when the index is positive was 75% and the negative predictive value or the probability that the inflammatory process is not present when the index is negative was 80%. Hence the C_4 _quotient method (Reibergram) was found to be superior in sensitivity, specificity and predictive values.

## Discussion

The complement system consists of more than thirty proteins and has three types of activation pathways: classical, lectin and alternative pathways. The complement system not only has a role in innate immunity but also works as an antibody-dependent effector to eliminate pathogens. In serum, normal or decreased levels of C_3 _and C_4 _are associated with specific immune-mediated diseases. In patients with immune-mediated diseases in the CNS, it is essential to measure complement activation in CSF and the intrathecal synthesis of complement proteins.

The immune response within the CNS has specific characteristics associated with a reduced immunological environment, being a unique and special compartment without much peripheral regulation. The CNS humoral immune response is different from the immune response observed in blood, mainly because there is no switch from the IgM class response to IgG or IgA class response in the CSF in the course of inflammatory neurological disease. The pattern of intrathecal IgG/IgA/IgM synthesis remains fairly constant and depends on the cause, pathophysiology and localization of the disease process [3,4]. In addition, it is known that there is a long-lasting but slow decay of intrathecal antibody synthesis, sometimes detectable up to 20 years after sufficient treatment (e.g. in neurosyphilis, neuroborreliosis or HSV-encephalitis). In CSF, there are polyclonal and polyspecific immune responses in diseases like multiple sclerosis [20,24]. Recent studies have demonstrated that the intrathecal synthesis of C_3c _is associated with infectious [14] and autoimmune disease [15].

Studies carried out on the passage of different blood proteins into the CSF have established different models that tried to explain their differential diffusion into the cerebral compartment. The theory of protein flux/CSF flow [4,24] suggests that there is no loss of selectivity during dysfunction of the blood-CSF barrier, and that the only variable determining the speed of passage of a blood protein into CSF is the coefficient of molecular diffusion which is inversely proportional to the molecular size of the protein. The protein concentrations in CSF depend on blood concentration, blood-CSF barrier function and intrathecal synthesis [25]. In order to know if a protein in CSF is brain-derived it should have at least one of the following characteristics: a higher CSF concentration than in serum, the blood-derived fraction in CSF significantly lower than brain-derived fraction, or if the coefficient of variation (CV) of the serum concentrations is larger than CV in CSF. In our experiment, the C_4 _concentration in blood was larger than in CSF in controls and in patients with meningoencephalitis. In the patient group, the CV in CSF was larger than in serum but in the control group, the CV was not larger in CSF than in blood. This suggests that although C_4 _is a blood-derived protein, during the inflammation process there was an increased amount produced in brain. This study showed that 75% of patients with meningoencephalitis had intrathecal C_4 _synthesis with or without dysfunction of the blood-CSF barrier as indicated by its QAlb value which varies according to age. In addition, the control group with variable QAlb values was in the normal range, indicating no intrathecal synthesis, which is consistent with the absence of cerebral inflammation in this group [26]. Also noteworthy is that none of the evaluated cases was placed below the lower hyperbolic curve, which is an area with no biological explanation. In medical practice, a technical error in protein quantification is suspected when a case falls in this area, so this is a quality control for the method.

Using the previously-used C_4_-Index to study intrathecal C_4_synthesis has disadvantages over the quotient method. The index is a linear formula whereas the distribution of proteins from blood to cerebral compartments follows a hyperbolic curve [1], thus the C_4_-Index may produce false results [25]. In addition, indexes can not be applied when there is a dysfunction of the blood-CSF barrier, which greatly limits its application in inflammatory diseases that usually involve barrier dysfunction. There is also a practical problem; the values of the index vary with the volume of extracted CSF since there is a rostro-caudal concentration gradient of proteins passing into CSF, and the first and the following volumes collected have different concentrations [27]. However, it has been demonstrated that since the Reibergram works with protein quotients normalized to quotient (CSF/serum) these are not affected by the extracted CSF volume [1,25,27].

Despite these limitations, the results of the C_4 _Reibergram were compared to those of the index since there is no other known gold standard. There was a positive significant statistical correlation between both methods. Nevertheless, these results demonstrated the superior diagnostic value of Reibergram.

## Conclusion

Under all conditions of the blood-CSF barrier, the CSF/Serum quotient graph can identify the occurrence of intrathecal C_4 _synthesis. It can quantify the C_4 _fraction which is locally produced in the CNS and differentiate from C_4 _that may have entered the CSF from blood. On the other hand, the C_4_-Index should not be used in patients with low QAlb and in particular with high QAlb, i.e. with blood-CSF barrier dysfunction.

## Competing interests

The authors declare that they have no competing interests.

## Authors' contributions

BPD and AJDC designed and coordinated the study and drafted the manuscript. RBCF participated in its design and reviewed the clinical profiles of patients. ARR helped with protein analysis and in drafting the manuscript. All authors read and approved the final version of the manuscript.
